# Neuronavigated shockwaves with low energy ameliorate cognitive deficits and depressive symptoms in Alzheimer’s disease

**DOI:** 10.1192/j.eurpsy.2026.10255

**Published:** 2026-07-31

**Authors:** U. Sprick, A. R. Guenes, M. Beglau

**Affiliations:** Center of neurostimulation, Alexius/Josef Clinic, Neuss, Germany

## Abstract

**Introduction:**

Alzheimer’s disease (AD) is still a very common cause of dementia and a common cause of death in elderly humans. No effective long-term treatment has been found so far.

**Objectives:**

TPS (Transcranial Pulse Stimulation) which can be individually tracked by MRI-scans offers new perspectives to ameliorate deficits caused by AD. Pilot studies show beneficial effects of TPS on learning and memory. There are also reports of restorative structural changes in the thickness of the cerebral cortex due to the stimulation.

**Methods:**

65 out-patients with Alzheimer’s disease (with light to moderate symptoms) received 6.000 pulses of TPS (0.2 mJ/mm^2^ per single pulse, with a frequency of 4 Hz) per session. The application of the pulses with Neurolith by Storz Medical was inividually navigated by use of current MRT-images of the patients. TPS-pulses were administered bilaterally into the frontal, parietal and temporal cortex. Pulses were applicated over a period of 2 weeks (3 sessions per week). Booster TPS-sessions were applied once every 6 weeks. Cognitive capabilities (especially executive functions) of the patients were tested using the Stroop-Test (colour-word-interference-test). The Stroop-Test is a standardized test for executive functions. Patients were tested using a pre – post design (t_0_ pre stimulation : t_1_ two weeks and 6 months later).

**Results:**

TPS-stimulation over a period of two weeks (6 sessions) showed ameliorating effects on performance in the Stroop-Test. The mean-score was diminished significantly (pre vs. post ; p < 0.05 – paired T-test). Single patients showed extraordinary improvements by shortening completer times in the Stroop-Test by halve. Mood and depressive symptoms of the patients were also ameliorated. The mean of the BDI measured before and after the treatment period of 2 weeks diminished from 19.2 to 11.5. The beneficial effects remained up to 6 months, No significant side-effects occured during all the sessions in none of the patients. In single cases patients complained about transient headaches (4 %) and fatigue (3 %).

**Conclusions:**

The results of this trial show that cognitive impairments of executive functions and depressive symptoms in Alzheimer’s disease may be ameliorated using TPS as a noninvasive brain stimulation method. No severe side-effects were observed.

Thus neuronavigated TPS may be considered as a novel promissing tool of noninvasive brain stimulation (NIBS) in severe neurodegenerative diseases like Alzheimer’s disease.

**Disclosure of Interest:**

None Declared

